# The genetics of low and high birthweight and their relationship with cardiometabolic disease

**DOI:** 10.1007/s00125-025-06420-8

**Published:** 2025-04-10

**Authors:** Gunn-Helen Moen, Liang-Dar Hwang, Caroline Brito Nunes, Nicole M. Warrington, David M. Evans

**Affiliations:** 1 Institute for Molecular Bioscience, The University of Queensland, Brisbane, QLD Australia; 2 Institute of Clinical Medicine, Faculty of Medicine, University of Oslo, Oslo, Norway; 3 Department of Public Health and Nursing, K.G. Jebsen Center for Genetic Epidemiology, Norwegian University of Science and Technology (NTNU), Trondheim, Norway; 4 The Frazer Institute, The University of Queensland, Woolloongabba, QLD Australia; 5 MRC Integrative Epidemiology Unit, University of Bristol, Bristol, UK

**Keywords:** Birthweight, Developmental origins of health and disease, Diabetes, DOHaD, Genome-wide association, GWAS

## Abstract

**Aims/hypothesis:**

Low birthweight infants are at increased risk not only of mortality, but also of type 2 diabetes mellitus and CVD in later life. At the opposite end of the spectrum, high birthweight infants have increased risk of birth complications, such as shoulder dystocia, neonatal hypoglycaemia and obesity, and similarly increased risk of type 2 diabetes mellitus and CVD. However, previous genome-wide association studies (GWAS) of birthweight in the UK Biobank have primarily focused on individuals within the ‘normal’ range and have excluded individuals with high and low birthweight (<2.5 kg or >4.5 kg). The aim of this study was to investigate genetic variation associated within the tail ends of the birthweight distribution, to: (1) see whether the genetic factors operating in these regions were different from those that explained variation in birthweight within the normal range; (2) explore the genetic correlation between extremes of birthweight and cardiometabolic disease; and (3) investigate whether analysing the full distribution of birthweight values, including the extremes, improved the ability to detect genuine loci in GWAS.

**Methods:**

We performed case–control GWAS analysis of low (<2.5 kg) and high (>4.5 kg) birthweight in the UK Biobank using REGENIE software (*N*_low_=20,947; *N*_high_=12,715; *N*_controls_=207,506) and conducted three continuous GWAS of birthweight, one including the full range of birthweights, one involving a truncated GWAS including only individuals with birthweights between 2.5 and 4.5 kg and a third GWAS that winsorised birthweight values <2.5 kg and >4.5 kg. Additionally, we performed bivariate linkage disequilibrium (LD) score regression to estimate the genetic correlation between low/normal/high birthweight and cardiometabolic traits.

**Results:**

Bivariate LD score regression analyses suggested that high birthweight had a mostly similar genetic aetiology to birthweight within the normal range (genetic correlation coefficient [*r*_G_]=0.91, 95% CI 0.83, 0.99), whereas there was more evidence for a separate set of genes underlying low birthweight (*r*_G_=−0.74, 95% CI 0.66, 0.82). Low birthweight was also significantly positively genetically correlated with most cardiometabolic traits and diseases we examined, whereas high birthweight was mostly positively genetically correlated with adiposity and anthropometric-related traits. The winsorisation strategy performed best in terms of locus detection, with the number of independent genome-wide significant associations (*p*<5×10^−8^) increasing from 120 genetic variants at 94 loci in the truncated GWAS to 270 genetic variants at 178 loci, including 27 variants at 25 loci that had not been identified in previous birthweight GWAS. This included a novel low-frequency missense variant in the *ABCC8* gene, a gene known to be involved in congenital hyperinsulinism, neonatal diabetes mellitus and MODY, that was estimated to be responsible for a 170 g increase in birthweight amongst carriers.

**Conclusions/interpretation:**

Our results underscore the importance of genetic factors in the genesis of the phenotypic correlation between birthweight and cardiometabolic traits and diseases.

**Graphical Abstract:**

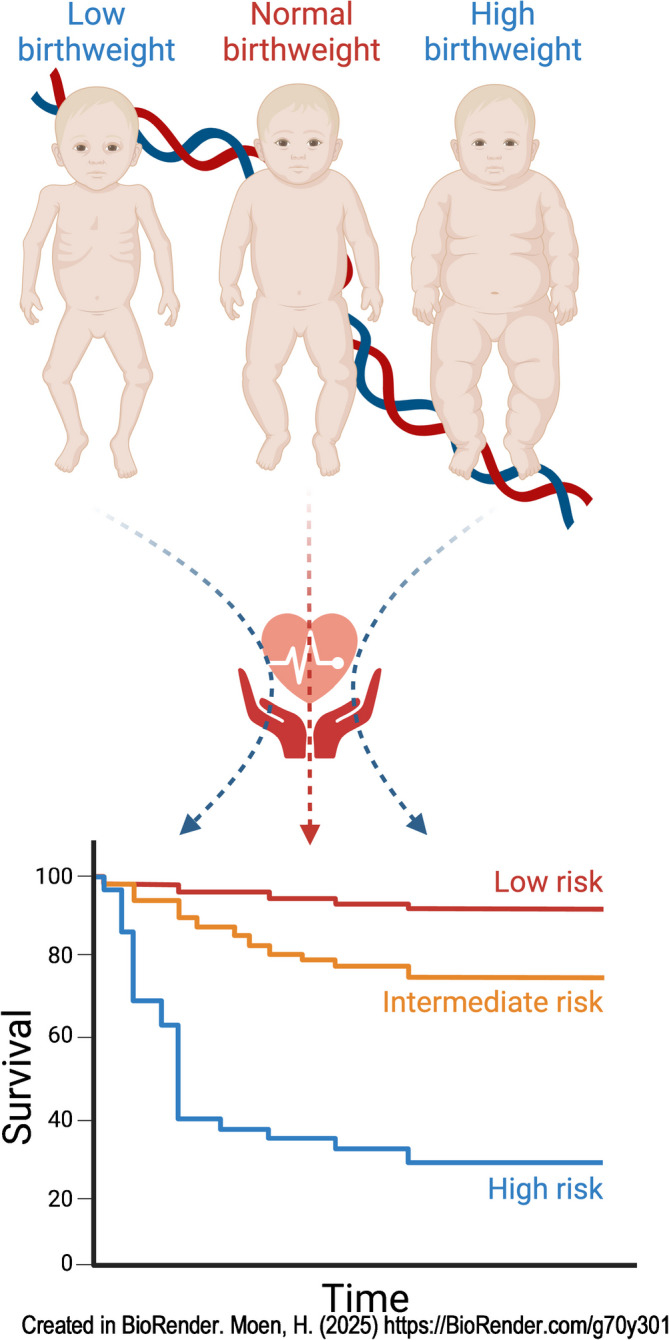

**Supplementary Information:**

The online version contains peer-reviewed but unedited supplementary material available at 10.1007/s00125-025-06420-8.


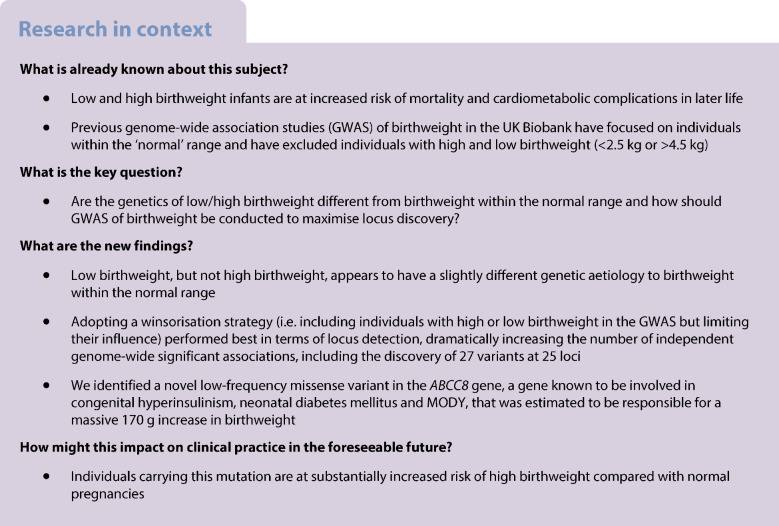


## Introduction

Low birthweight is associated with lower survival rate perinatally and increased risk of many chronic diseases in later life. Maternal undernutrition during pregnancy is one cause of low birthweight, which in turn is observationally associated with increased future risk of type 2 diabetes and CVD in offspring. The Forsdahl–Barker hypothesis and the developmental origins of health and disease (DOHaD) hypothesis provide an explanation for these robust observational associations [1–7]. In short, these hypotheses stipulate that impaired fetal growth and development in utero lead to developmental compensations, which programme the offspring to increased risk of disease in later life [5].

At the other end of the spectrum, children with high birthweight have an increased risk of birth complications, such as shoulder dystocia, neonatal hypoglycaemia and obesity, as well as increased long-term risk of developing metabolic syndrome, type 2 diabetes and CVD [8, 9]. The current obesity epidemic raises concerns that overnutrition in utero, together with obesity and high glucose levels in the mother, could lead to permanent metabolic changes in the fetus [10, 11]. Given the rapidly increasing incidence of cardiometabolic disease in many populations, understanding the aetiology of the relationship between birthweight and cardiometabolic disease is likely to be useful in terms of predicting whether intervening on prenatal factors is likely to yield useful reductions in disease risk.

Large-scale genome-wide association studies (GWAS) have identified over 243 loci associated with birthweight and provided seminal insights into its genetic aetiology and relationship with other common complex traits and diseases [12–17]. A 2016 GWAS meta-analysis suggested that the negative phenotypic correlation between birthweight and cardiometabolic disease was primarily mediated by genetic factors [13]. This finding is important because the Forsdahl–Barker and DOHaD hypotheses have emphasised the causal role of environmental factors in the genesis of these relationships. However, the 2016 results are not necessarily inconsistent with environmental hypotheses, since the authors employed strict data cleaning protocols including a decision to exclude individuals who had values for birthweight outside the ‘normal’ range, i.e. <2.5 kg and >4.5 kg [13]. Therefore, the results from the 2016 paper (and subsequent Early Growth Genetics [EGG] Consortium GWAS [16] papers) may only apply to birthweight within the normal range and not generalise to the tail ends of the distribution. In other words, it is possible that the genetic and environmental factors that underlie extremes of birthweight may be different from those that are responsible for variation within the normal range of the distribution.

The authors’ justification for the exclusion criteria in the 2016 paper was twofold: first, to identify loci whose primary effect was on birthweight, rather than, for example, gestational duration. This was important because some cohorts, such as the UK Biobank (UKBB) [18], did not record information on gestational age. Second, outlying scores for birthweight are expected to have disproportionate effects on the results of GWAS. The inclusion of these individuals could therefore decrease power to detect loci (e.g. if the reason for the outlying score is environmental rather than genetic).

We would argue, however, that there is good reason to consider including individuals with birthweight measurements that fall outside the normal range in GWAS. First, if there is interest in elucidating the relationship between birthweight and cardiometabolic disease, then it is important to include individuals who have extreme birthweights and are likely to be most informative for the hypotheses under investigation. Second, genuine, but outlying, values may increase power to detect association if the trait values reflect genetic factors that also operate in the remainder of the distribution. For example, previous GWAS of other anthropometric phenotypes including BMI have shown that including outlying individuals can increase power to detect common variants that operate across the entire phenotypic distribution [19]. Finally, even if individuals with, for example, very low birthweight are different from others in the distribution, it is important to demonstrate this empirically. Indeed, the identification of genetic variants that proxy extreme values could be useful in terms of future Mendelian randomisation studies to investigate the relationship between environmental factors that influence this part of the birthweight distribution and cardiometabolic disease.

The aim of the present study was threefold: (1) to investigate the genetic overlap between low birthweight (<2.5 kg), high birthweight (>4.5 kg) and birthweight within the normal range (birthweight between 2.5 kg and 4.5 kg); (2) to estimate SNP-based heritability (*h*_SNP_^2^) and the genetic correlations between low/high birthweight and later life traits including cardiometabolic phenotypes; and (3) to explore which of three GWAS strategies is likely to perform better in terms of locus detection when analysing continuous birthweight data, i.e. a strategy including all individuals in the GWAS regardless of whether their birthweight was extreme, the current strategy adopted by the EGG Consortium of performing a truncated GWAS excluding individuals with extreme birthweight (birthweight <2.5 kg or >4.5 kg) or a GWAS that winsorises extreme birthweight values.

## Methods

The UKBB is a large, prospective, population-based cohort containing ~500,000 individuals (approximately 273,000 women), with a variety of phenotypic and genome-wide genetic data available [20].

We used imputed genetic data from the October 2019 (version 3) release of the UKBB for our analyses (Application ID: 53641). The quality control was performed centrally by the UKBB. We excluded individuals who we did not identify as ancestrally white British based on *k*-means clustering applied to the first four genetic principal components generated by the UKBB and projected into the 1000 Genomes Project [21] space, as previously described [22]. We also excluded individuals who had withdrawn their consent to participate in the study as of February 2021.

### Birthweight

Participants in the UKBB reported their own birthweight. Individuals were excluded from analyses if they reported being part of a multiple birth. A total of 241,168 individuals had birthweight data measured in kilograms available. We grouped individuals as having low birthweight if their birthweight was less than 2.5 kg, as having a normal birthweight if their birthweight was between 2.5 kg and 4.5 kg and as having high birthweight if their birthweight was more than 4.5 kg. These cut-off values were based on previous publications that excluded individuals from birthweight GWAS analyses if they were classified as having unusually low or high birthweight [13, 16].

### Genome-wide association analysis

We ran two case–control GWAS analyses of the autosomal chromosomes (low birthweight vs normal birthweight, and high birthweight vs normal birthweight; electronic supplementary material [ESM] Table 1) using REGENIE software version 3.0.3 [23] (https://rgcgithub.github.io/regenie/). We also ran three GWAS of (continuous) self-reported birthweight using BOLT-LMM version 2.4 [24] (https://hsph.harvard.edu/research/price-lab/software/), one containing all individuals with birthweight data (i.e. no exclusion of outlying individuals, *N*=241,168 individuals), one involving individuals with birthweights in the normal range of 2.5–4.5 kg only (*N*=207,506 individuals) and a third GWAS where we winsorised the distribution of birthweights (i.e. any individual with a birthweight <2.5 kg was recorded as 2.5 kg, and any individual with a birthweight >4.5 kg was recorded as 4.5 kg; *N*=241,168 individuals). For all GWAS, we included sex, year of birth, genotyping batch and five genome-wide genetic principal components (as generated by the UKBB) as covariates. Variants were analysed as dosages, and those with minor allele frequency (MAF) less than 0.1%, or an imputation info score of less than 0.4, were excluded. Since we were interested in which strategy was most effective in terms of locus detection, we performed a look-up of confirmed birthweight-associated variants from the deCODE GWAS [17] in our continuous GWAS and compared the empirical results across the different strategies.

### Post-GWAS analyses

Independent genome-wide significant SNP signals were defined using standard settings in FUMA (https://fuma.ctglab.nl/) (i.e. 250 kb window size between linkage disequilibrium [LD] blocks, *r*^2^ thresholds of 0.6 and 0.1 to define independent SNPs within and between LD blocks and genome-wide significance threshold of *p*=5×10^−8^) [25]. FUMA was also used to annotate results and for locus zoom plots. An LD reference map for white British individuals in the UKBB embedded in FUMA was used for all analyses. The intercepts from univariate LD score regression analyses [26, 27] were used to investigate whether genomic inflation was likely due to polygenicity or population stratification/cryptic relatedness.

All the lead independent genome-wide significant SNPs discovered in our GWAS analysis were looked up in the deCODE [17] and the EGG GWAS [16] of birthweight along with both a maternal [28] and a fetal [29] GWAS of gestational age. Those variants not reaching genome-wide significance were further checked to see whether they were within ±500 kb of a genome-wide significant variant (*p*<5×10^−8^) in either of the two previous birthweight GWAS. Variants that were not identified in the previous GWAS were deemed to represent novel loci.

We used the GWAS atlas [30] (https://atlas.ctglab.nl/) to perform look-ups of top hits from the GWAS analyses. We used a genome-wide significant threshold of 5×10^−8^ to determine whether our top hits had any previous links with birthweight or other phenotypes. In order to investigate whether our loci primarily reflected genetic associations with birthweight (as opposed to gestational age), we performed multi-trait conditional and joint analysis (mtCOJO) [31] of genome-wide significant birthweight SNPs, conditioning on the maternal GWAS of gestational age [28, 29]. We used the maternal GWAS in this analysis because gestational age is primarily influenced by the maternal genome and consequently there are comparatively few known SNPs in the offspring genome robustly associated with the trait [28].

### Genetic correlations

To estimate genetic correlations between traits, we used bivariate LD score regression as implemented in the CTG-VL platform [32] (https://vl.genoma.io/). All summary result statistics from the birthweight GWAS were uploaded to the server and SNP heritability and genetic correlations were calculated. CTG-VL uses pre-computed LD scores amongst HapMap 3 SNPs from a European population provided by the original developers of LD score regression [26, 27], with the MHC region excluded. We estimated the genetic correlation between our two case–control GWAS and a range of phenotypes listed in ESM Table 2, including the EGG birthweight GWAS [16] and the three continuous birthweight GWAS run in this paper.

### Ethics approval and consent to participate

The UKBB has ethical approval from the North West Multi-centre Research Ethics Committee, which covers the UK, and all participants provided written informed consent. This project received ethical approval from the Institutional Human Research Ethics Committee, University of Queensland (Approval Number 2019002705).

#### Sex and gender

This manuscript used genetics to determine sex at birth used in the analysis.

## Results

### Birthweight phenotype in the UKBB

Self-reported birthweights in the UKBB ranged from 0.45 kg to 8.00 kg, with a mean of 3.35 kg and a standard deviation of 0.65 kg. When we restricted the range of birthweight from 2.50 kg to 4.50 kg, the mean birthweight was 3.39 kg with a standard deviation of 0.42 kg. ESM Fig. 1 shows the distribution of birthweights using these two selection schemes. Both distributions were approximately normal (assessed by eye), with the full sample showing a right skew with birthweight values as large as 8 kg.

### GWAS of birthweight in the UKBB

ESM Figs 2–6 show Q–Q plots and genomic inflation factors for each of the GWAS (λ between 1.11 and 1.43; ESM Table 3). However, LD score regression intercepts (ESM Table 3) were all between 1.02 and 1.12, suggesting that the majority of the inflation in the λ scores was due to genuine polygenic signals. The GWAS of winsorised birthweight was the only GWAS with an LD score intercept above 1.1, consistent with a slight inflation. Manhattan plots for all GWAS can be found in ESM Figs 7–11. Variants at 15 loci reached genome-wide significance across the two case–control GWAS (ESM Table 4, ESM Figs 7, 8). This small number of significant loci contrasts with the high numbers detected in the continuous GWAS (see below). In addition, SNPs at all but one of the loci in the case–control GWAS had been previously associated with birthweight, suggesting that dichotomising the phenotype added little in terms of locus discovery compared with continuous GWAS, although a handful of loci did attain lower *p* values in the high birthweight GWAS (ESM Table 4). The lead SNP at the one novel locus from the high vs normal birthweight GWAS (*p*=9.9×10^−9^), rs67254669, is a physically genotyped, low-frequency (MAF=0.001) missense variant in the *ABCC8* gene. This SNP was also significantly associated with birthweight in two of the continuous birthweight GWAS, when we included all individuals (β=0.199 kg per addition of the minor G allele, SE=0.026, *p*=4.2×10^−14^) and when we winsorised the distribution (β=0.170 kg per addition of the minor allele, SE=0.022, *p*=1.3×10^−14^), and had a very large effect size. No other variant in the region reached genome-wide significance, potentially due to its low frequency and lack of LD with surrounding markers. Nevertheless, the low-frequency allele also showed nominally significant association with increased risk of gestational diabetes mellitus and type 2 diabetes in publicly available FinnGen data [33] (gestational diabetes: logistic β=0.619 per addition of the minor G allele, SE=0.166, *p*=2×10^−4^; type 2 diabetes: logistic β=0.299 per addition of the minor G allele, SE=0.070, *p*=2×10^−5^), and decreased (inverse rank normal transformed) glucose levels (*p*=1.9×10^−4^), but not type 2 diabetes, HbA_1c_ or offspring birthweight (all *p*>0.05), in publicly available GWAS summary statistics from the UKBB published on the Neale website (https://www.nealelab.is/). The variant was not available in the publicly available deCODE summary results GWAS statistics for birthweight.

**Fig. 1:**
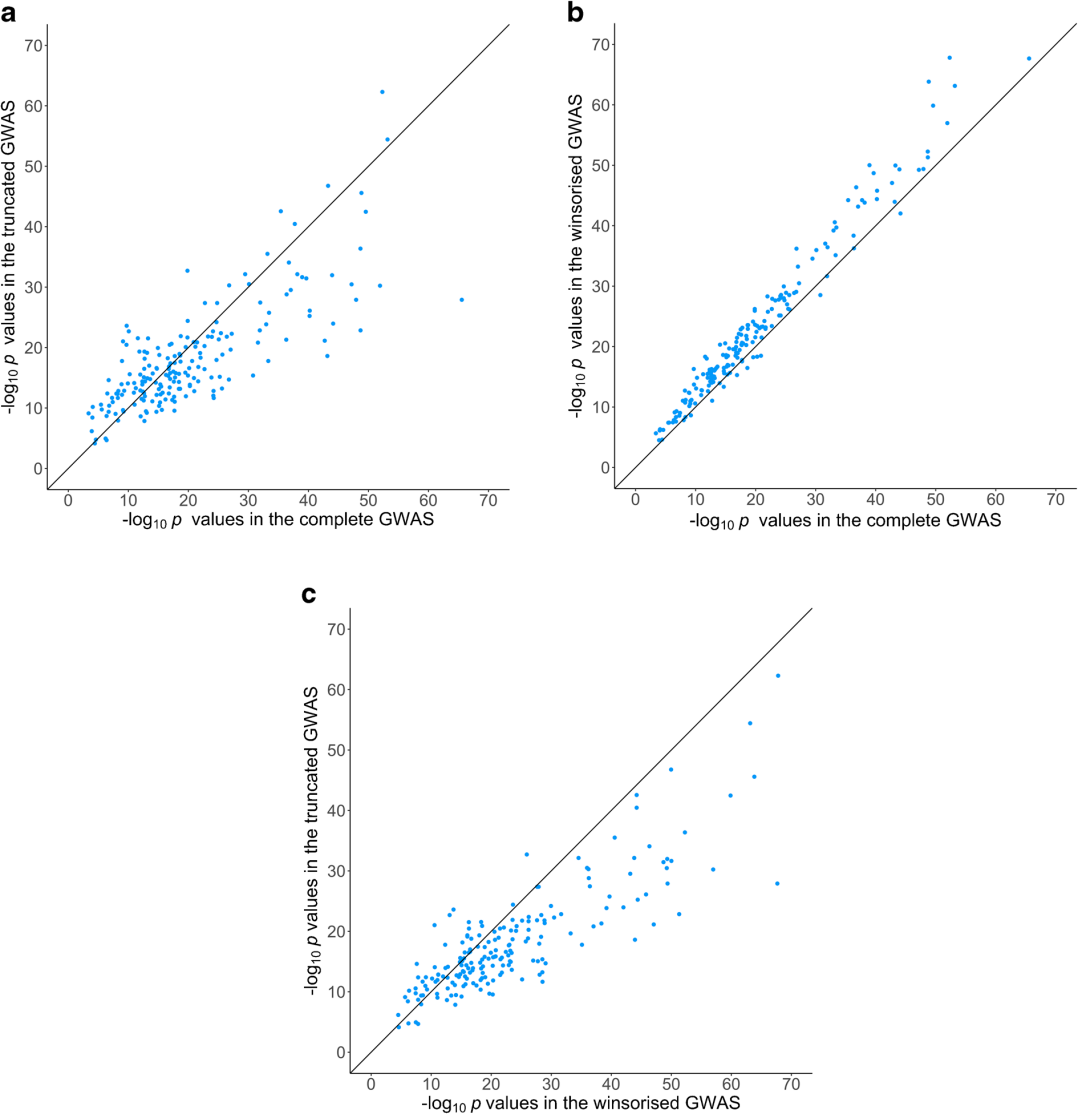
The −log_10_ *p* values of genome-wide significant SNPs from the deCODE GWAS of own birthweight in the full birthweight GWAS vs the truncated GWAS (**a**), the full birthweight GWAS vs the winsorised GWAS (**b**) and the truncated GWAS vs the winsorised GWAS (**c**). In (**a**), 117 SNPs had lower *p* values in the full birthweight GWAS and 79 had lower *p* values in the truncated GWAS. In (**b**), 180 SNPs had lower *p* values in the winsorised GWAS and 16 SNPs had lower *p* values in the full GWAS. In (**c**), 150 SNPs had lower *p* values in the winsorised GWAS and 46 SNPs had lower *p* values in the truncated GWAS

Figure 1 presents −log_10_ *p* values from the three continuous GWAS in the present study for 196 SNPs robustly associated with birthweight, which were previously identified/confirmed in the deCODE study and present in the current study [17]. The graphs clearly show that the EGG Consortium strategy of performing GWAS on the truncated distribution of birthweight reduces the signal at these known variants on average. This is despite the likely presence of ‘winner’s curse’ in the selection of the 196 variants (i.e. the deCODE paper used EGG Consortium data where the distribution of birthweight in the UKBB was truncated, and so variant selection is biased towards those variants that do well in the truncated GWAS), which is likely reflected in the more similar performance of the strategies in that part of the *p* value distribution close to the cut-off for genome-wide significance (where the effect of winner’s curse will be greatest). In contrast, winsorisation performed the best on average amongst the three strategies in terms of maximising the signal at these known loci. The implication is that the winsorising strategy is also likely to perform better in terms of identifying novel loci. Consequently, we focus on presenting the results from these analyses in the main part of the paper.

The GWAS of the winsorised birthweight distribution resulted in 270 lead SNPs at 178 loci reaching genome-wide significance (ESM Table 5, ESM Fig. 9), compared with only 120 lead SNPs at 94 loci when analysing birthweights between 2.5 and 4.5 kg (ESM Table 6, ESM Fig. 10), and 186 lead SNPs at 143 loci when analysing the full distribution of birthweights (ESM Table 7, ESM Fig. 11) (there were also a small number of SNPs that were significant in the truncated GWAS/full sample but that were not significant in the winsorised sample). This included 27 variants that were not within ±500 kb of a SNP reaching *p*<5×10^−8^ in the previous EGG or deCODE birthweight GWAS (Table 1, ESM Table 8, ESM Figs 12–38). Of the 27 variants at these new loci, we note that nine of the SNPs had stronger evidence of association in the larger deCODE study (compared with the truncated UKBB results), six had less strong evidence and 12 were not reported. Additionally, several have been previously associated with cardiometabolic and/or anthropometric phenotypes at genome-wide levels of significance, and so represent good candidates for genuine associations with birthweight (ESM Table 8). Interesting variants include those in *ABCC8* (discussed above) and a variant in a long non-coding RNA that contains antisense instructions for the gene *SLC16A1*. The robustness of all these associations will need to be confirmed in future GWAS.

**Table 1:** Novel loci identified in the continuous GWAS analysis using the winsorisation of birthweight method

Variant	Nearest gene	Chromosome	Position (bp)	Effect allele	MAF	β	SE	*p* value	Imputation info
1:113501899_AGGT_A	*SLC16A1-AS1*	1	113501899	AGGT	0.229	0.0109	0.0018	2.30×10^−09^	0.989
rs78444298	*EDEM3*	1	184672098	G	0.015	0.0303	0.0055	3.60×10^−08^	1.000
2:111875799_GA_G	*ACOXL*	2	111875799	GA	0.467	−0.0094	0.0015	1.20×10^−09^	0.963
rs111864601	*CUL3*	2	225388104	T	0.453	0.0089	0.0016	9.10×10^−09^	0.959
rs11709779	*KLF7P1*	3	170649012	C	0.360	−0.0091	0.0016	7.10×10^−09^	1.000
rs2291714	*AREGB*	4	75485813	C	0.314	−0.0100	0.0018	3.80×10^−08^	0.879
rs538957912	*ARRDC3*	5	90574984	C	0.150	0.0132	0.0022	1.60×10^−09^	0.959
6:41909521_CCG_C	*CCND3*	6	41909521	CCG	0.092	0.0180	0.0031	9.10×10^−09^	0.886
rs7748510	*CGA*	6	87784311	G	0.236	0.0108	0.0018	4.60×10^−09^	0.981
6:88049043_CGTGT_C	*SMIM8*	6	88049043	CGTGT	0.398	−0.0095	0.0017	9.20×10^−09^	0.870
rs760277758	*CHN2*	7	29340089	AT	0.305	0.0101	0.0017	1.50×10^−09^	0.985
rs6462990	*SUGCT*	7	40913646	T	0.410	0.0091	0.0015	4.10×10^−09^	0.989
rs11375604	*A1CF*	10	52646867	G	0.203	0.0122	0.0020	1.20×10^−09^	0.971
rs4757634	*PARVA*	11	12596885	A	0.446	−0.0087	0.0016	2.40×10^−08^	0.993
rs67254669	*ABCC8*	11	17470143	A	0.004	−0.1695	0.0220	1.30×10^−14^	0.999
rs528647	*BUD13*	11	116523821	C	0.114	−0.0148	0.0025	5.90×10^−09^	0.968
11:122500489_CT_C	*UBASH3B*	11	122500489	CT	0.142	0.0137	0.0023	3.00×10^−09^	0.965
rs11222084	*ADAMTS8*	11	130273230	A	0.330	0.0087	0.0016	3.00×10^−08^	1.000
rs35681675	*DLEU1*	13	51130374	T	0.411	0.0086	0.0016	4.90×10^−08^	0.981
rs12889267	*ARHGEF40*	14	21542766	A	0.133	0.0122	0.0020	2.00×10^−09^	1.000
rs759112030	*ARHGEF40*	14	21555087	AAATC	0.246	−0.0089	0.0016	3.30×10^−08^	0.917
rs369279418	*SLC9A5*	16	67300812	CT	0.048	0.0223	0.0038	2.90×10^−09^	0.856
rs9921675	*C16orf95*	16	87270478	A	0.295	−0.0095	0.0017	3.20×10^−08^	0.995
rs145763145	*SMG6*	17	2152185	C	0.344	0.0101	0.0016	8.30×10^−10^	0.968
rs61750863	*CCNE1*	19	30312976	A	0.004	0.0726	0.0131	3.10×10^−08^	1.000
rs200876443	*PTOV1:AC018766.4*	19	50363585	CAG	0.041	−0.0226	0.0041	2.70×10^−08^	1.000
rs35704817	*IFNGR2*	21	34769729	G	0.036	−0.0227	0.0041	4.00×10^−08^	1.000

One of the reasons for excluding extreme birthweight measurements was to avoid detecting loci that were primarily associated with gestational age rather than birthweight. In the case of the dichotomous GWAS, we found that SNPs at three genome-wide significant loci exhibited nominal associations with the maternal and/or fetal GWAS of gestational age (*p*<0.05, variants at *ADCY5* [both], *AMZ1:GNA12* [maternal] and *LINC00880* [fetal]) (ESM Table 4). For the winsorised GWAS of birthweight, we found that 24 of the sentinel genome-wide significant SNPs were also nominally associated with own gestational age and 38 with maternal gestational age (5×10^−8^<*p*<0.05; ESM Table 5), including one (at *RP11-542A14.1*) that was also genome-wide significant in the maternal gestational age GWAS. Of the 27 variants at loci detected with the winsorisation method and deemed to be novel (Table 1, ESM Table 8), 11 were available for analysis with mtCOJO. Most of these SNPs showed a slight attenuation in their *p* value compared with the birthweight GWAS; however, evidence for association with birthweight remained strong.

### Genetic correlations

**Fig. 2:**
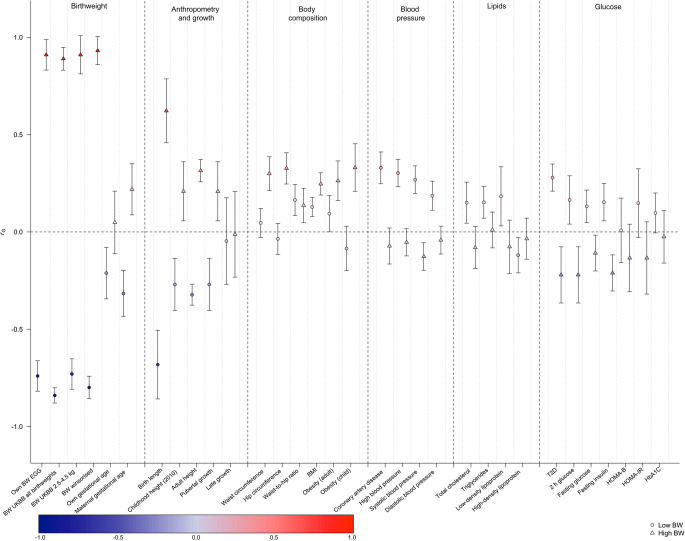
Genetic correlation (*r*_G_) between either high (triangles) or low (circles) birthweight and cardiometabolic-related phenotypes. The colour scale represents the strength of genetic correlation from −1 (dark blue) to 1 (dark red). A genetic correlation of exactly zero would be shown as white. BW, birthweight; T2D, type 2 diabetes

We performed bivariate LD score regression analyses to investigate the degree of genetic similarity between low birthweight, high birthweight and birthweight within the normal range (i.e. from the truncated birthweight GWAS). We found that high birthweight was strongly genetically correlated with birthweight within the normal range (genetic correlation coefficient [*r*_G_]: 0.91; 95% CI 0.83, 0.99; Fig. 2, ESM Table 9), whereas the magnitude of the genetic correlation between low birthweight and birthweight in the normal range was slightly lower (*r*_G_: −0.74; 95% CI 0.66, 0.82; Fig. 2, ESM Table 9). In addition, the low birthweight trait exhibited an increased SNP-based heritability (*h*_SNP_^2^) compared with the other traits (*h*_SNP_^2^=0.26 for low birthweight, *h*_SNP_^2^=0.03 for high birthweight, *h*_SNP_^2^=0.11 for both all birthweights and winsorised birthweight and *h*_SNP_^2^=0.08 for truncated birthweight) (ESM Table 3), despite fewer loci reaching genome-wide significance. Low birthweight was moderately positively genetically correlated with many cardiometabolic traits (coronary artery disease, type 2 diabetes, systolic and diastolic blood pressure etc.), whereas high birthweight showed mostly low, non-significant negative genetic correlations with the same traits and positive genetic correlations with adiposity and anthropometric traits (height, BMI, obesity, waist and hip circumference etc.) (Fig. 2, ESM Table 10).

## Discussion

Previous GWAS meta-analyses of birthweight including the UKBB have removed individuals with birthweights <2.5 kg and >4.5 kg. The rationale has been that these individuals may be qualitatively different and consequently that their inclusion may decrease statistical power. Neither the results of our genetic correlation analyses nor the results of our empirical GWAS support this strategy. Rather, we found that winsorising the birthweight distribution dramatically increased the total number of genome-wide significant loci detected and enhanced the signals at known birthweight loci, compared with analysing a truncated distribution or analysing the complete distribution of birthweight. These results suggest that winsorising the distribution of birthweight may offer a good compromise between (accidentally) incorporating outlying measurements that reflect data entry errors/extreme environments, which lower power to detect association, and including genuine values that contain disproportionate levels of signal and increase statistical power. All our novel loci require replication in larger samples before they can be confirmed as genuine. However, the identification of variants that are robustly associated with phenotypes related to birthweight (e.g type 2 diabetes, blood pressure etc.) and for which there is additional evidence from the larger deCODE study argues strongly that at least a proportion of our new loci are likely to be real.

Amongst the novel loci identified in the winsorised GWAS of birthweight was a low-frequency missense variant in the *ABCC8* gene. Heterozygous individuals carrying this mutation were estimated to be on average 170 g heavier at birth compared with individuals homozygous for the common allele. Variants in *ABCC8* are known to cause several monogenic forms of diabetes, including permanent [34–36] and transient forms of neonatal diabetes mellitus [35], and familial hyperinsulinaemic hypoglycaemia [37, 38]. Likewise, common variants in the gene have been robustly associated with type 2 diabetes [39, 40] and height [41], amongst other complex traits. The *ABCC8* gene codes for the sulfonylurea receptor 1 protein, which is one subunit of the ATP-sensitive potassium (K-ATP) channel that is found across cell membranes of pancreatic beta cells. This channel controls the secretion of insulin into the bloodstream. Indeed, sulfonylureas, a class of oral glucose-lowering drugs used in the treatment of non-insulin-dependent diabetes mellitus, interact with this receptor to modify the conductance of the associated ion channels. We speculate that this new missense mutation may lead to hyperinsulinaemia and excessive growth, as insulin is a strong growth factor for the fetus.

An auxiliary aim of our study was to investigate whether the inclusion of individuals with extreme birthweight increased the likelihood of identifying loci whose primary associations reflected gestational age (i.e. rather than birthweight). Except for loci at *RP11-542A14.1*, the majority of birthweight-associated variants (and none of the new birthweight variants identified in the complete GWAS) did not show strong associations with gestational age in previous GWAS. Part of the reason for this could be differences in sample size between the birthweight and gestational age meta-analyses [28, 29]. However, conditional analyses using mtCOJO suggested that, for many loci, gestational age was unlikely to be a major mediator of the SNP–birthweight association. Given that several statistical methods exist for removing the (genetic) contribution of secondary traits to the primary GWAS [31, 42], we contend it makes little sense to truncate the distribution of birthweight because of concerns in identifying gestational age-related loci.

The results of LD score regression analyses were also consistent with the above claims, suggesting that the genetic aetiology of low and high birthweight was largely overlapping with birthweight in the middle part of the distribution. Whilst our cut-offs for what constitutes ‘low’ and ‘high’ birthweight are to some degree arbitrary (and their choice will consequently affect estimates of *h*_SNP_^2^ and genetic correlation), there was some evidence for a separate set of genes being important for low birthweight (*r*_G_=−0.74; 95% CI 0.66, 0.82; for low birthweight compared with normal birthweight as defined by the EGG Consortium [16]). Although the *h*_SNP_^2^ of low birthweight was higher than many of the other traits (*h*_SNP_^2^=0.26), the GWAS of low vs normal birthweight control participants resulted in a paucity of genome-wide significant associations and no new variants being discovered. It is unclear why this is the case but it could happen if, for example, the variants influencing low birthweight were of smaller effect and/or in the low-frequency part of the spectrum, and so there was not enough power to reach genome-wide significance, but still enough signal to contribute to genome-wide estimates of *h*_SNP_^2^.

Our genetic correlation analyses showed substantial genetic correlations between low birthweight and several cardiometabolic traits and diseases, including coronary artery disease, blood pressure and type 2 diabetes. These results are similar to and complement the results of both individual level [13] and summary results [13, 16] genetic correlation analyses between birthweight in the normal part of the distribution (between 2.5 kg and 4.5 kg) and cardiometabolic traits. One possibility is that genetic pleiotropy forms part of the explanation for the inverse phenotypic correlation between birthweight and cardiometabolic disease, and consequently an alternative explanation for the Forsdahl–Barker hypothesis [43] and potentially DOHaD more broadly. The corollary is that Mendelian randomisation studies that purport to investigate the relationship between birthweight and cardiometabolic disease/traits need to take into account the possibility of horizontal pleiotropy as a possible explanation for positive results [44–48].

The results in the present manuscript relate specifically to birthweight. However, the broader question about whether to include individuals with extreme phenotypes in GWAS more generally is also important. Previous GWAS of extreme phenotypes (e.g. obesity) have shown that individuals at the ends of the trait distribution can be informative for identifying common variants of small effect that affect the entire distribution of values [19]. In other words, for at least some phenotypes, individuals have extreme values not only because of rare variants and/or extreme environments, but also because of the polygenic contribution of common variants. Individuals in this part of the distribution also typically exert the largest contribution on test statistics, meaning that their inclusion can have disproportionate effects on power to detect association at genuine loci. However, these individuals can also add noise to an analysis, particularly if their values represent data entry errors and/or contain significant amounts of measurement error.

In conclusion, we recommend that future GWAS of birthweight do not truncate the distribution before analysis and that a winsorisation strategy instead might be advantageous in terms of locus discovery. Using this approach, we found evidence for several new birthweight loci, including a low-frequency variant in the *ABCC8* gene of large effect. We also found evidence to suggest that low birthweight may have a genetic aetiology that is partially distinct from other parts of the birthweight distribution. Our results highlight the genetic links between birthweight and future risk of cardiometabolic disease.

## Supplementary Information

Below is the link to the electronic supplementary material.

ESM Figures (PDF 3670 KB)

ESM Tables (XLSX 155 KB)

## Data Availability

Human genotype and phenotype data from the UK Biobank on which the results of this study were based were accessed with accession ID 53641. The genotype and phenotype data are available upon application to the UK Biobank (http://www.ukbiobank.ac.uk/). Requirements for data access to the UK Biobank are described at https://www.ukbiobank.ac.uk/.

## Abbreviations

DOHaD: Developmental origins of health and disease
EGG: Early Growth Genetics
GWAS: Genome-wide association study
*h*_SNP_^2^: SNP-based heritability
LD: Linkage disequilibrium
MAF: Minor allele frequency
mtCOJO: Multi-trait conditional and joint analysis
*r*_G_: Genetic correlation coefficient
UKBB: UK Biobank
