# Supplementary material for: The genetics of low and high birthweight and their relationship with cardiometabolic disease
Source: Diabetologia. 2025 Apr 10;68(7):1452–62. doi: 10.1007/s00125-025-06420-8 (PMC12176956; doi:10.1007/s00125-025-06420-8)
Supplement: Supplementary file 1 — ESM Figures (PDF 3670 KB) [file 125_2025_6420_MOESM1_ESM.pdf]

Supplementary Materials

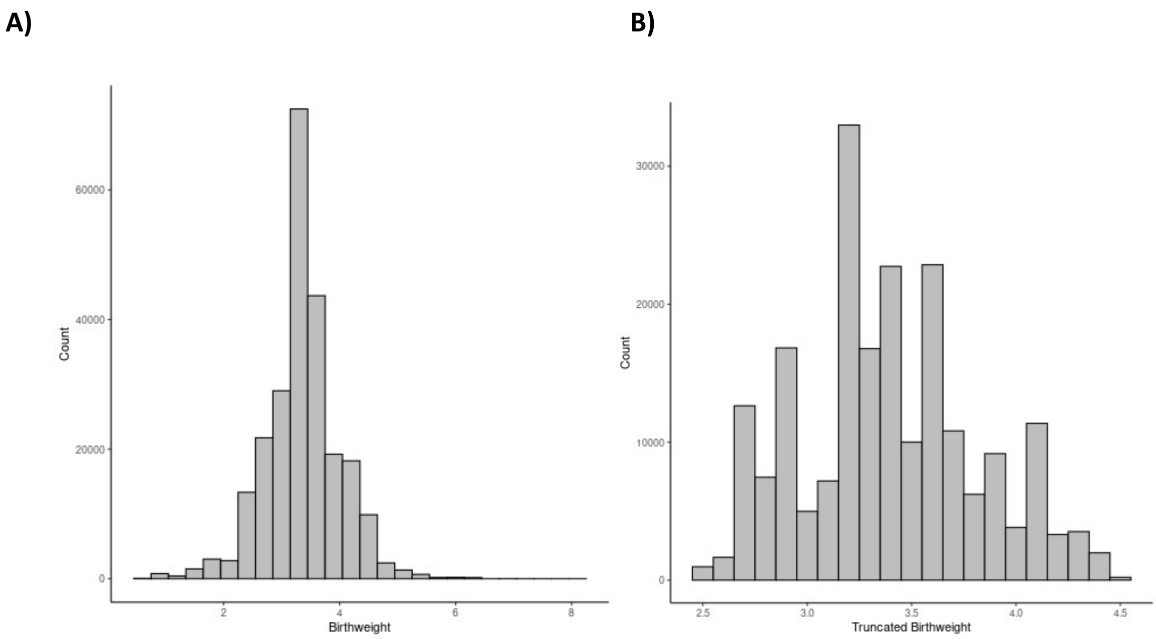

ESM Fig. 1: Distribution of birthweight in the UK Biobank. Panel A) shows the distribution of birthweights in the full UK Biobank sample and Panel B) shows the distribution of birthweights when the extremes are removed.

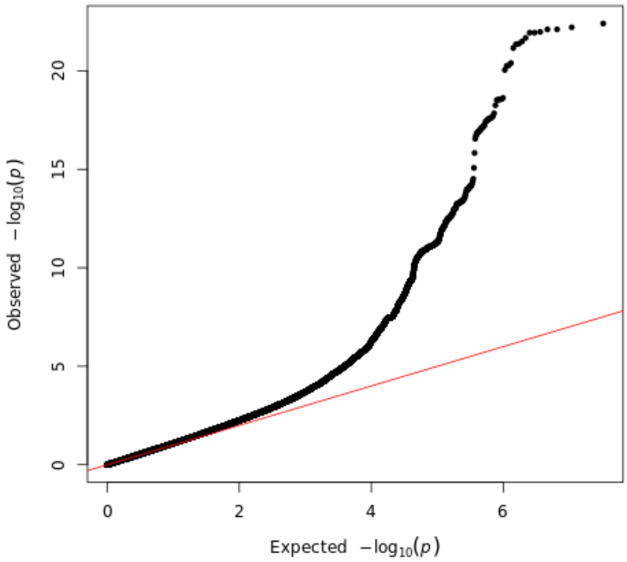

ESM Fig. 2: QQ plot of low versus normal birthweight. Genomic inflation factor (Lambda) = 1.17.

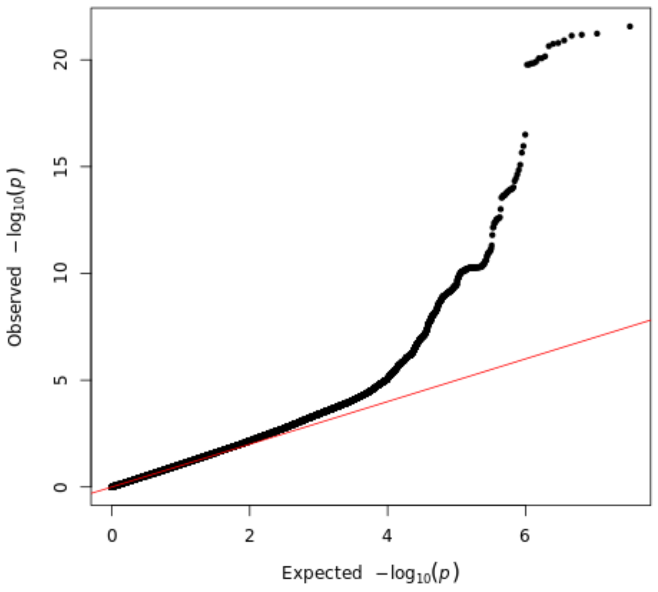

ESM Fig. 3: QQ plot of high versus normal birthweight. Genomic inflation factor (Lambda) = 1.11.

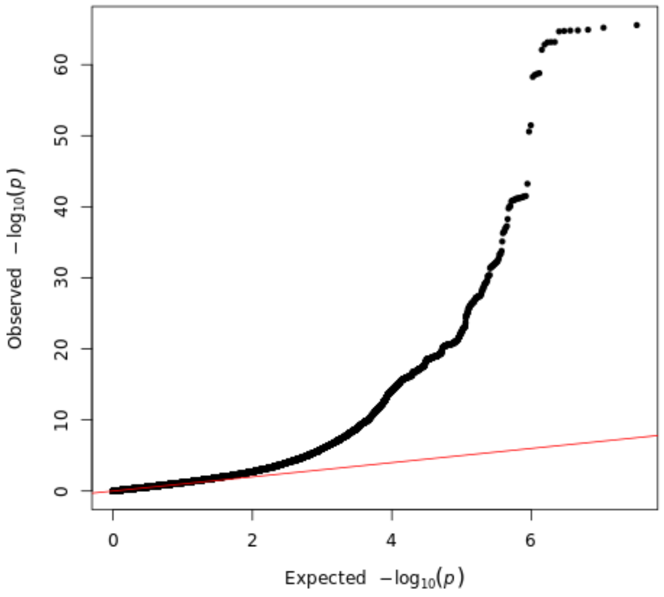

ESM Fig. 4: QQ plot of the GWAS of continuous birthweight. Genomic inflation factor (Lambda) = 1.37.

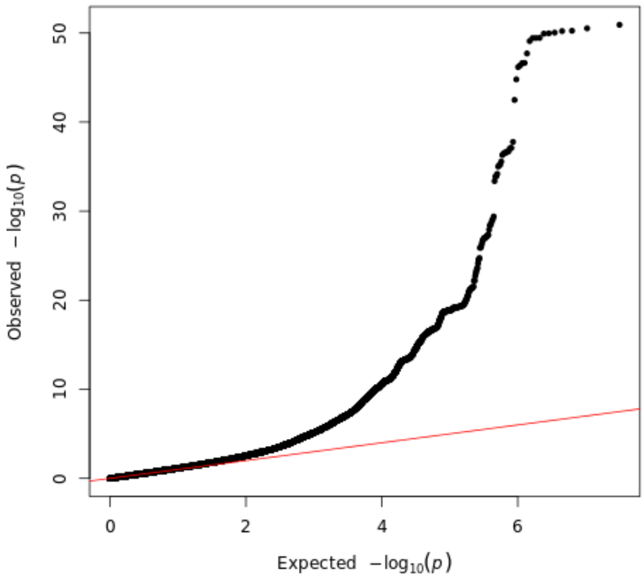

ESM Fig. 5: QQ plot of the GWAS of continuous birthweight between 2.5 and 4.5 kg. Genomic inflation factor (Lambda) = 1.31.

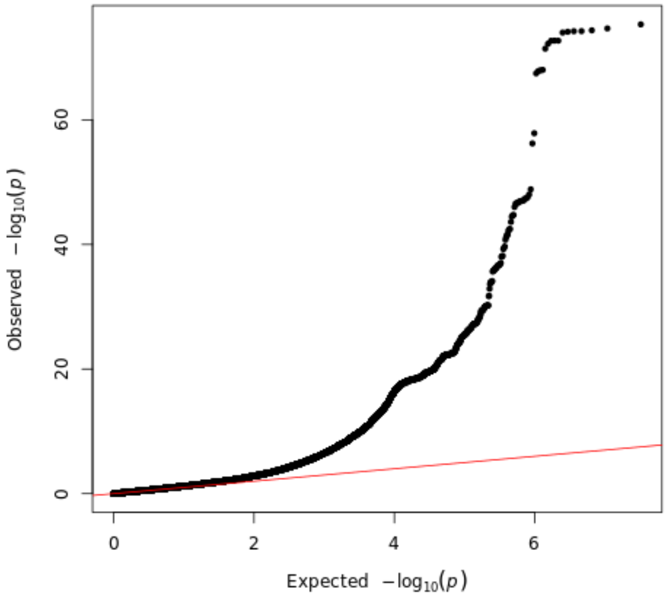

ESM Fig. 6: QQ plot of the GWAS of winsorized birthweight. Genomic inflation factor (Lambda) = 1.20.

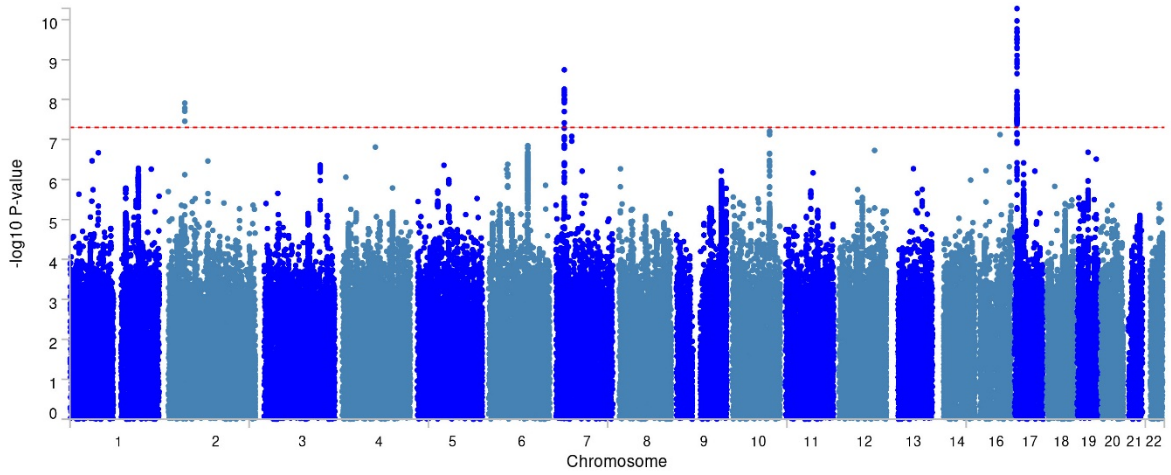

ESM Fig. 7: Manhattan plot of the GWAS of low birthweight versus normal birthweight.

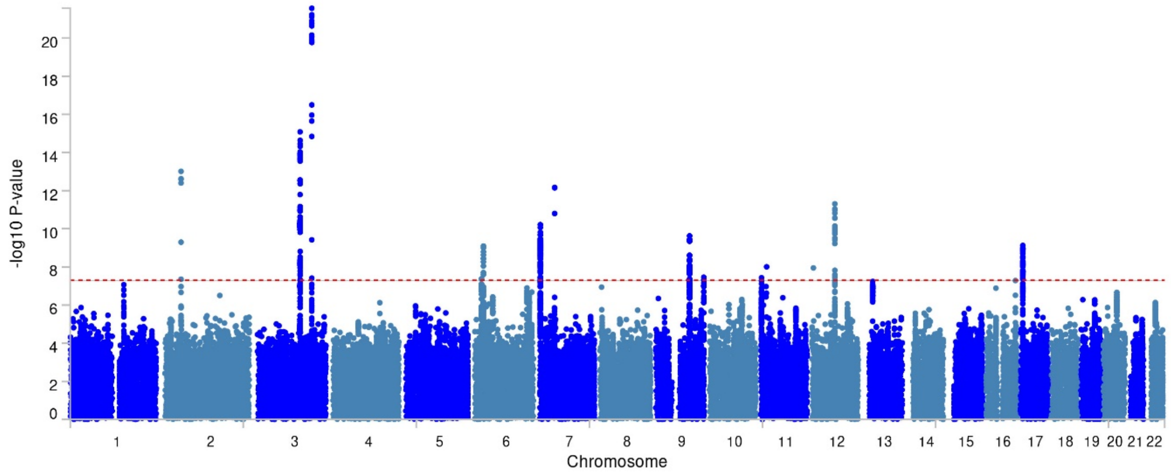

ESM Fig. 8: Manhattan plot of the GWAS of high birthweight versus normal birthweight.

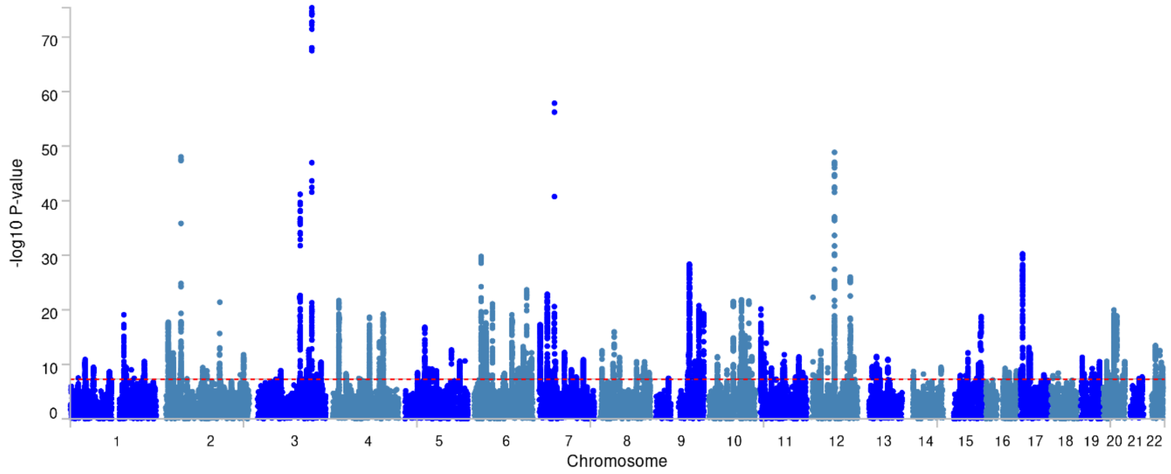

ESM Fig. 9: Manhattan plot of the GWAS containing winsorized birthweight values.

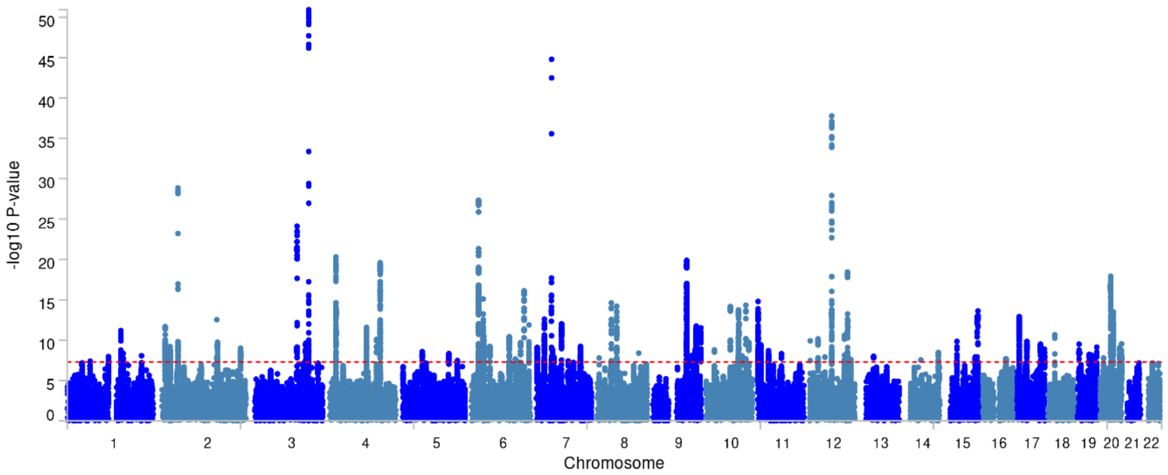

ESM Fig. 10: Manhattan plot of the GWAS containing truncated birthweight values between 2.5 and 4.5kg.

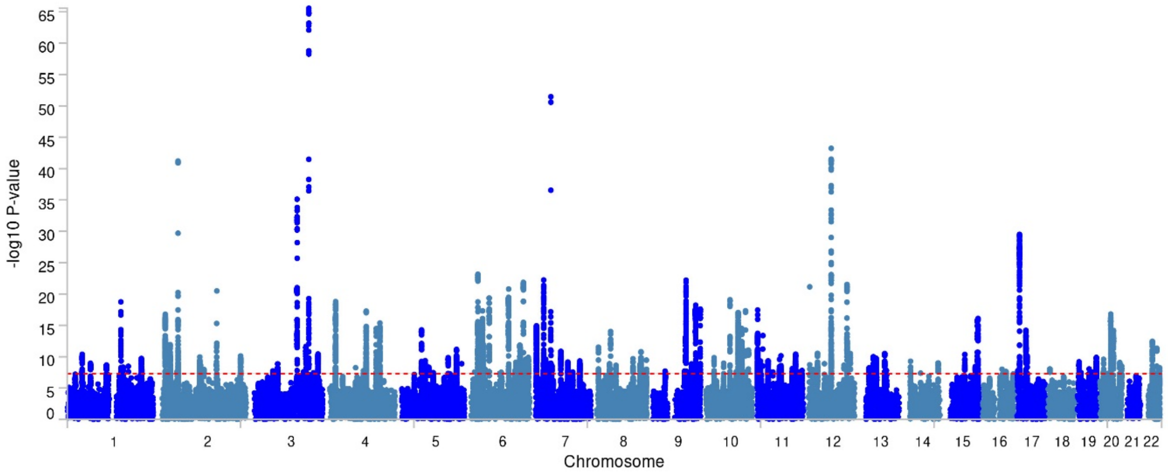

ESM Fig. 11: Manhattan plot of the GWAS containing the full distribution of birthweight values.

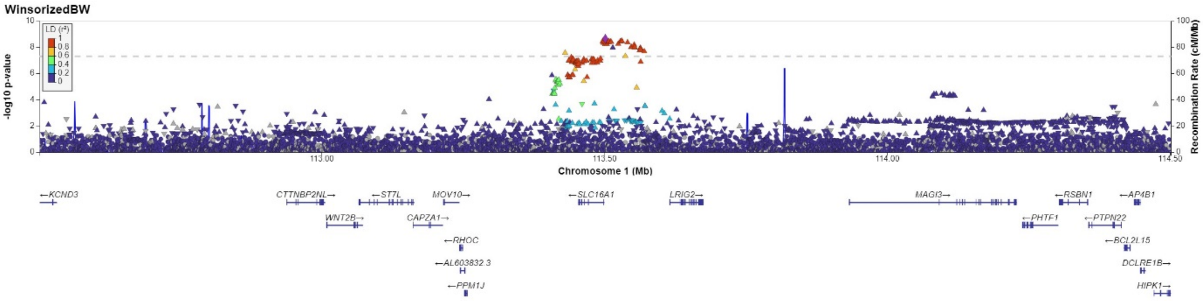

ESM Fig. 12: Locus zoom plot for 1:113501899_AGGT_A. SNPs which are not in the European reference panel are coloured grey.

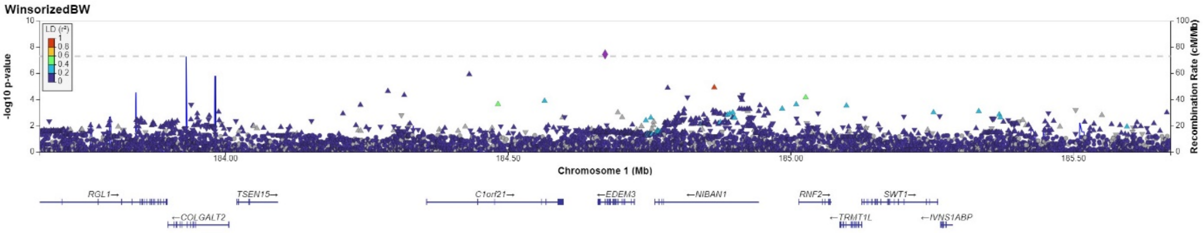

ESM Fig. 13: Locus zoom plot for rs78444298. SNPs which are not in the European reference panel are coloured grey.

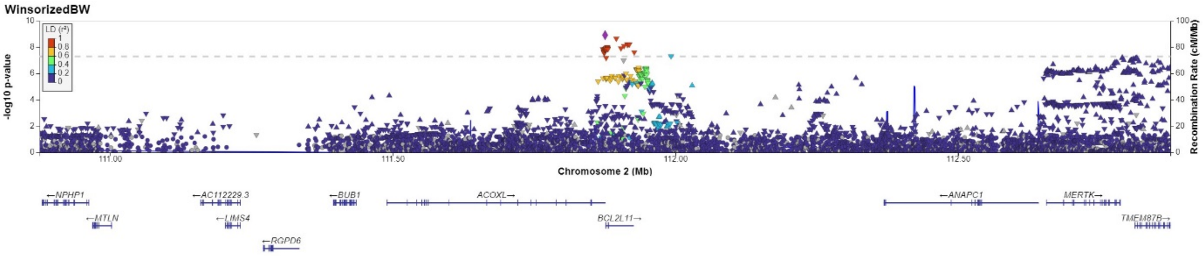

ESM Fig. 14: Locus zoom plot for 2:111875799_GA_G. SNPs which are not in the European reference panel are coloured grey.

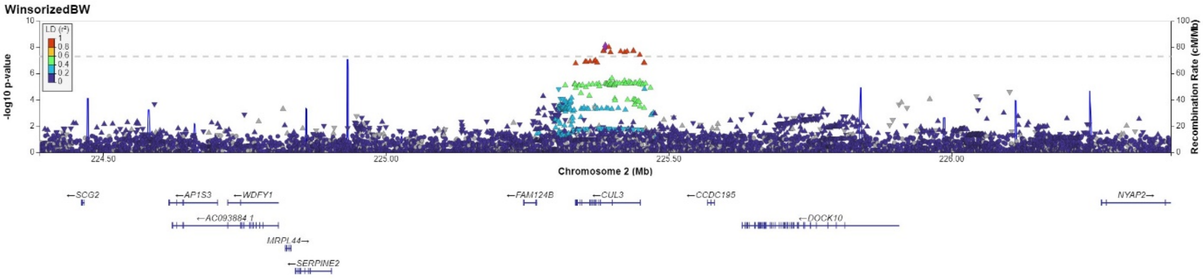

ESM Fig. 15: Locus zoom plot for rs111864601. SNPs which are not in the European reference panel are coloured grey.

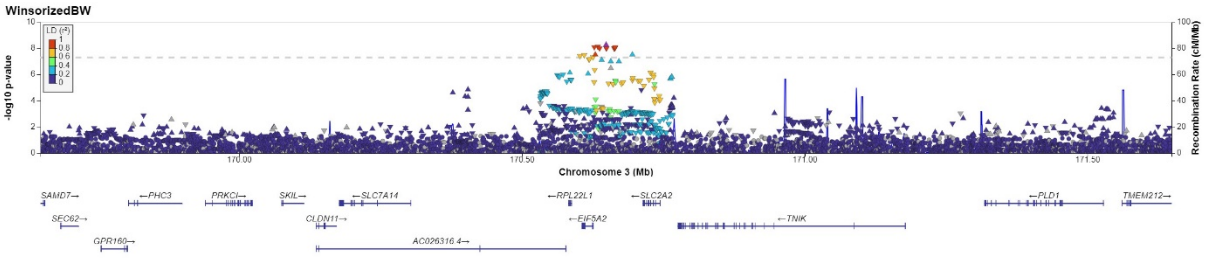

ESM Fig. 16: Locus zoom plot for rs11709779. SNPs which are not in the European reference panel are coloured grey.

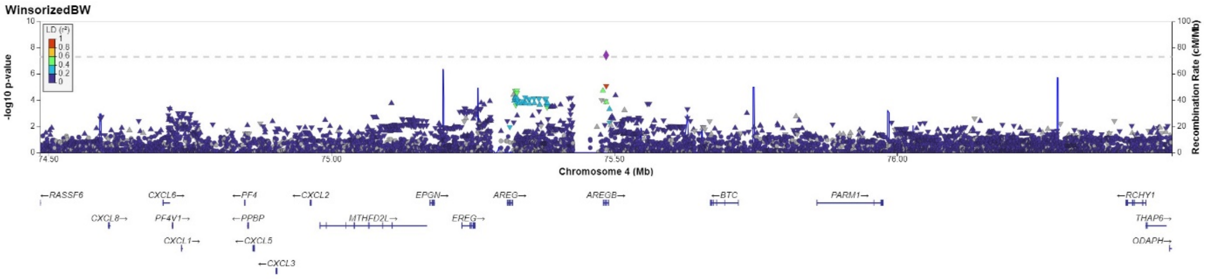

ESM Fig. 17: Locus zoom plot for rs2291714. SNPs which are not in the European reference panel are coloured grey.

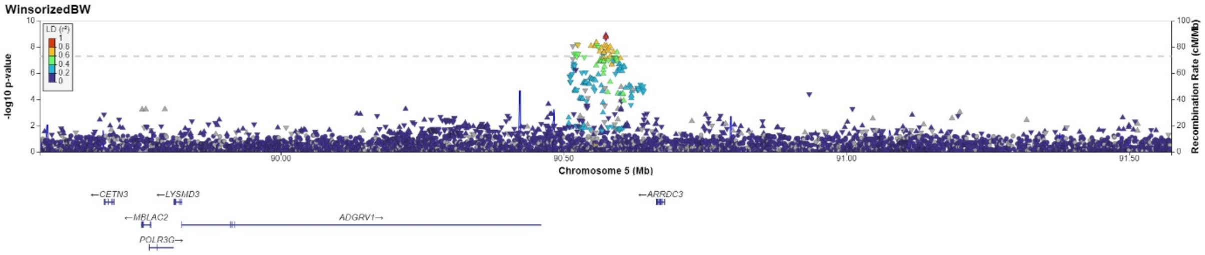

ESM Fig. 18: Locus zoom plot for rs538957912. SNPs which are not in the European reference panel are coloured grey.

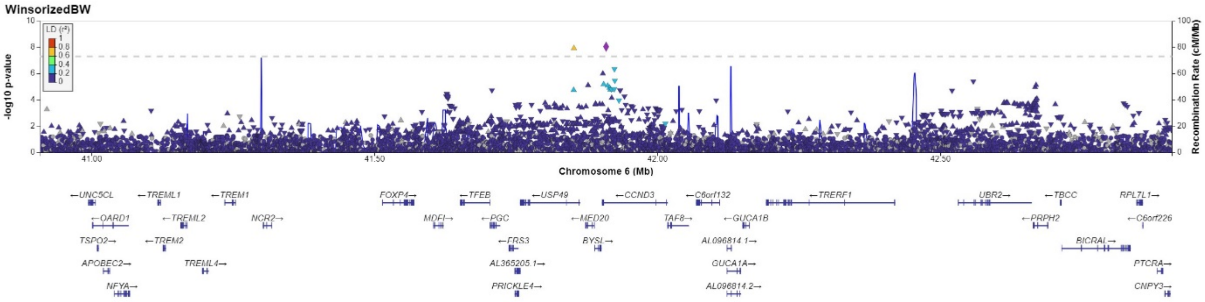

ESM Fig. 19: Locus zoom plot for 6:41909521_CCG_C. SNPs which are not in the European reference panel are coloured grey.

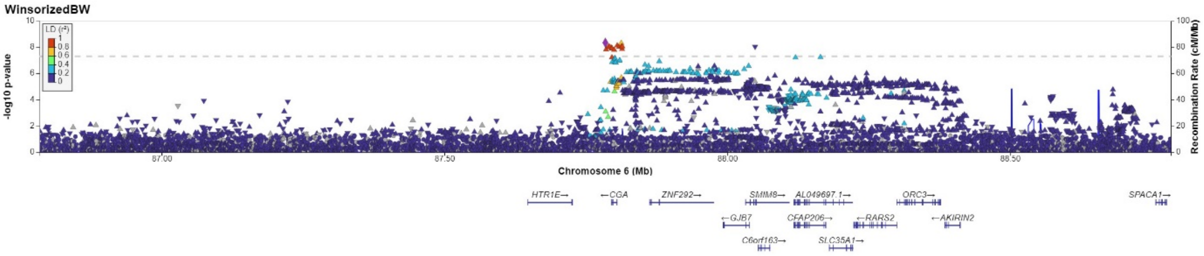

ESM Fig. 20: Locus zoom plot for rs7748510 SNPs which are not in the European reference panel are coloured grey.

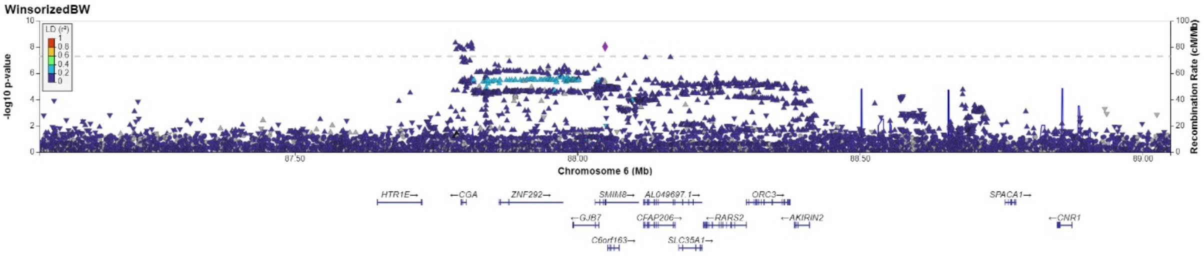

ESM Fig. 21: Locus zoom plot for 6:88049043_CGTGT_C. SNPs which are not in the European reference panel are coloured grey.

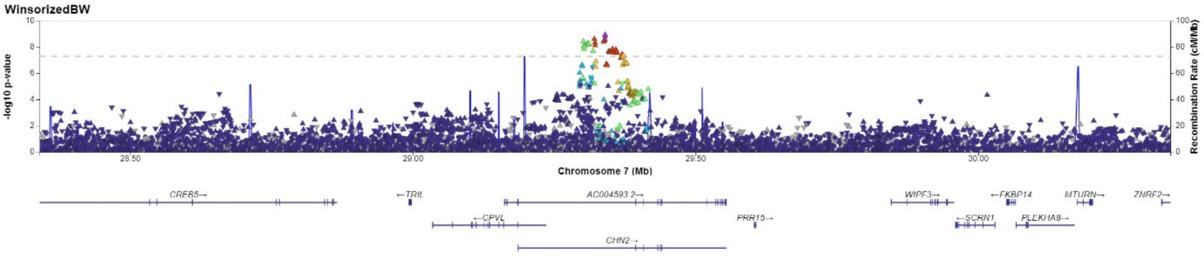

ESM Fig. 22: Locus zoom plot for rs760277758. SNPs which are not in the European reference panel are coloured grey.

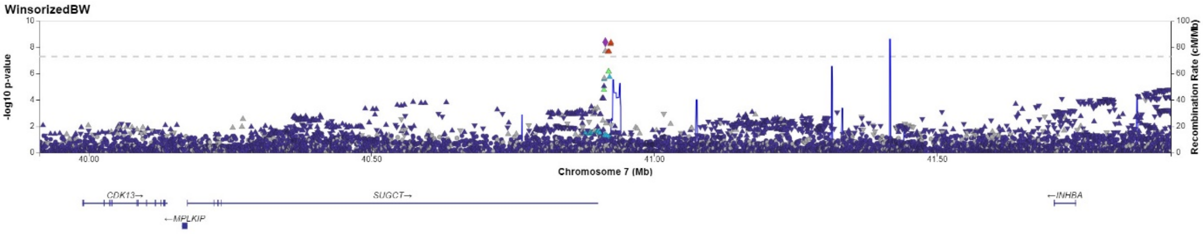

ESM Fig. 23: Locus zoom plot for rs6462990. SNPs which are not in the European reference panel are coloured grey.

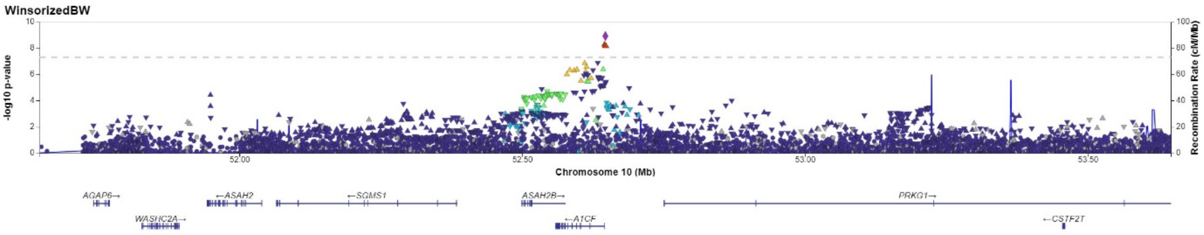

ESM Fig. 24: Locus zoom plot for rs11375604. SNPs which are not in the European reference panel are coloured grey.

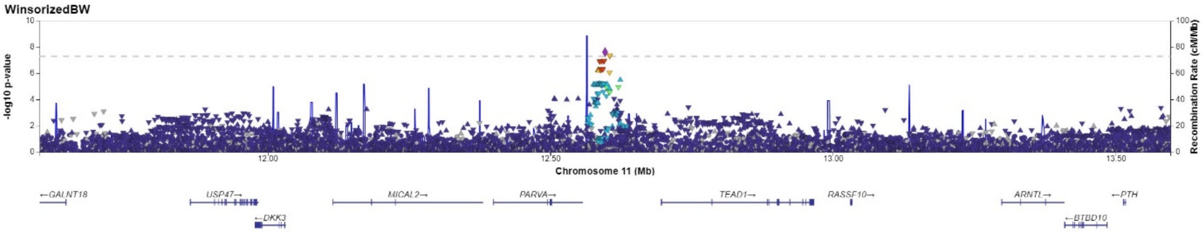

ESM Fig. 25: Locus zoom plot for rs4757634. SNPs which are not in the European reference panel are coloured grey.

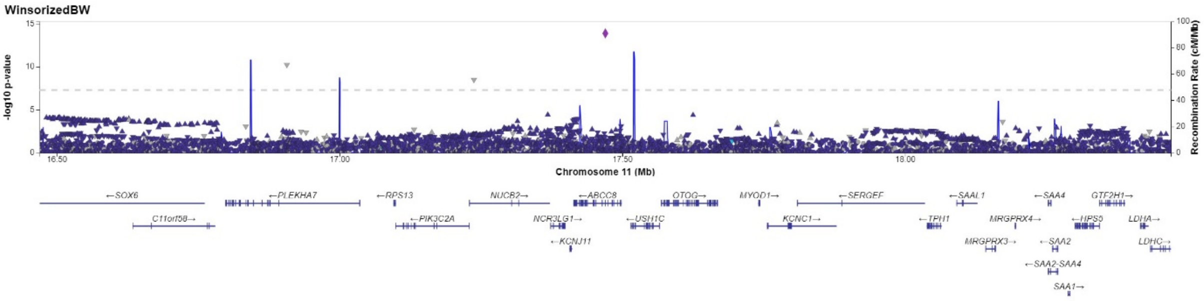

ESM Fig. 26: Locus zoom plot for rs67254669. SNPs which are not in the European reference panel are coloured grey.

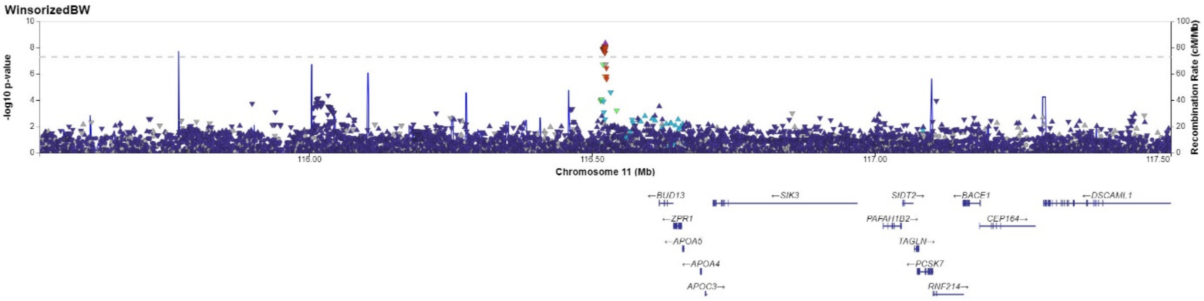

ESM Fig. 27: Locus zoom plot for rs528647. SNPs which are not in the European reference panel are coloured grey.

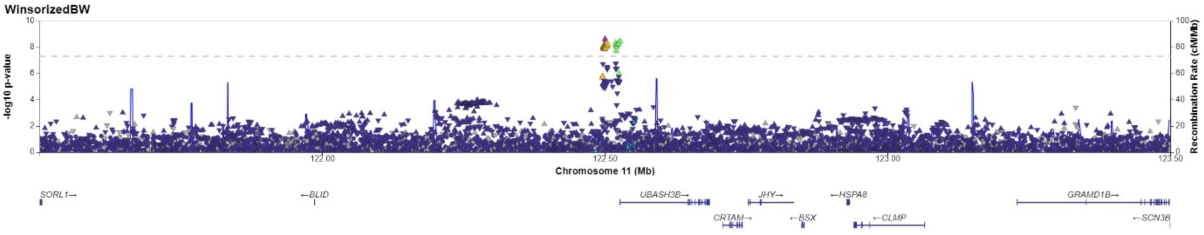

ESM Fig. 28: Locus zoom plot for 11:122500489_CT_C. SNPs which are not in the European reference panel are coloured grey.

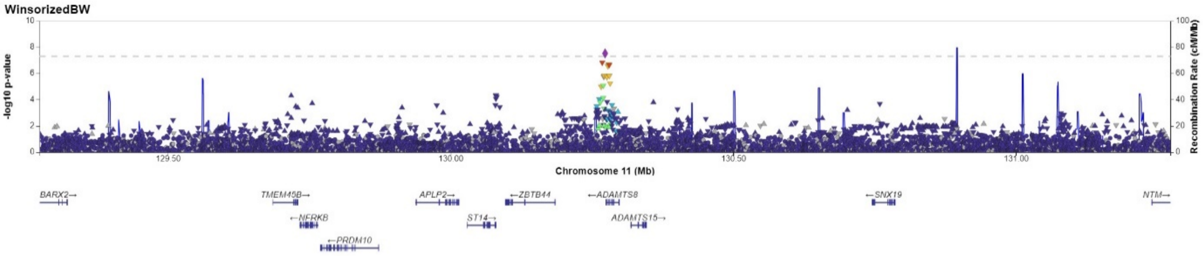

ESM Fig. 29: Locus zoom plot for rs11222084. SNPs which are not in the European reference panel are coloured grey.

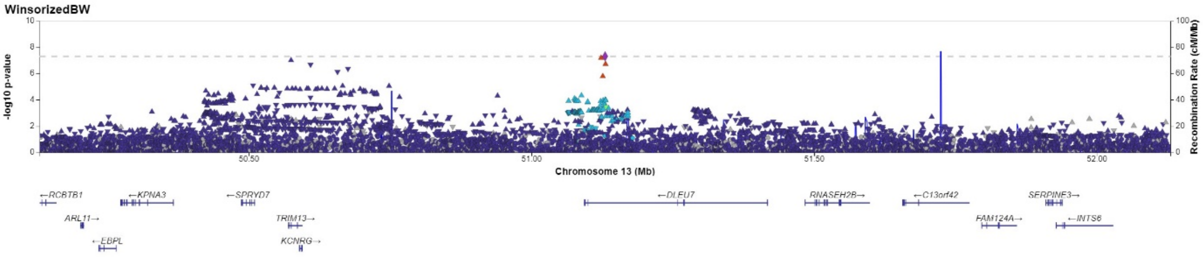

ESM Fig. 30: Locus zoom plot for rs35681675. SNPs which are not in the European reference panel are coloured grey.

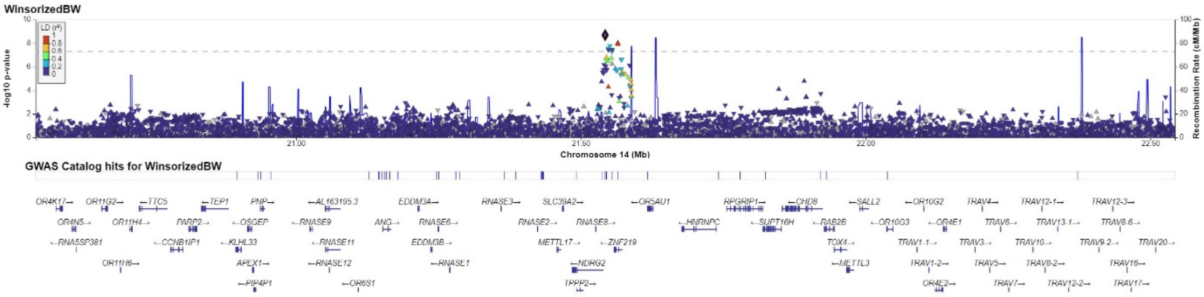

ESM Fig. 31: Locus zoom plot for rs12889267. SNPs which are not in the European reference panel are coloured grey.

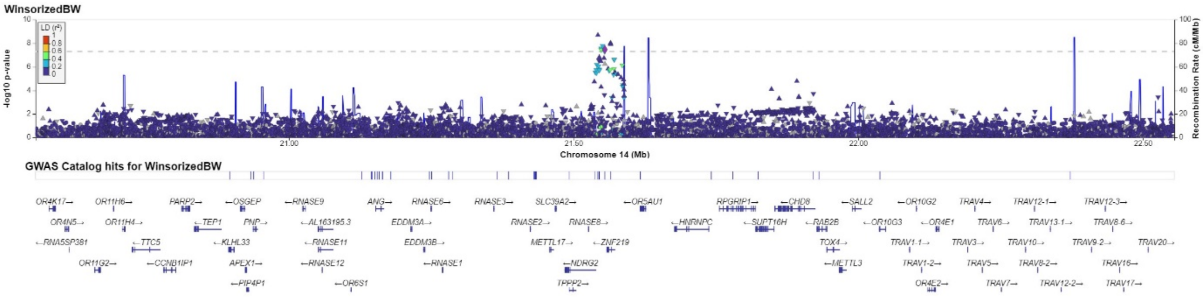

ESM Fig. 32: Locus zoom plot for rs759112030. SNPs which are not in the European reference panel are coloured grey.

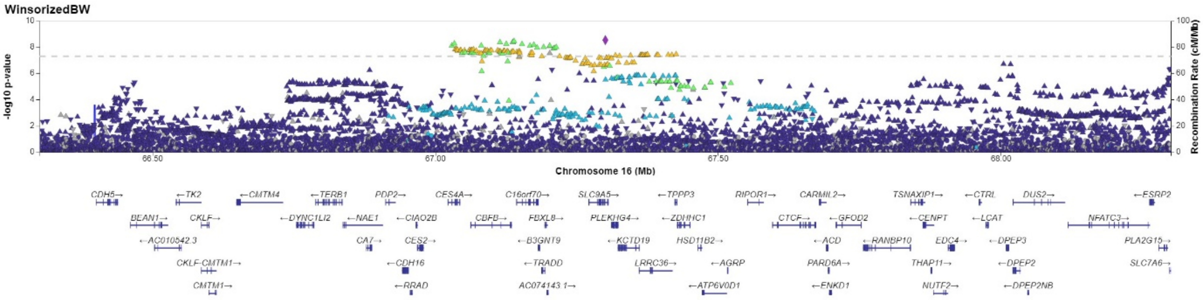

ESM Fig. 33: Locus zoom plot for rs369279418. SNPs which are not in the European reference panel are coloured grey.

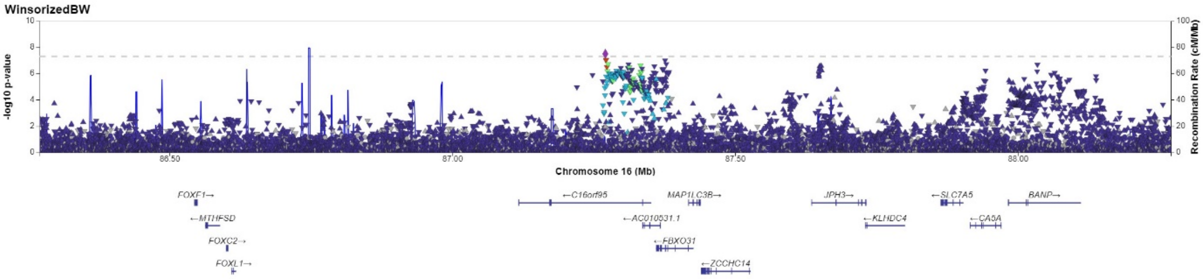

ESM Fig. 34: Locus zoom plot for rs9921675. SNPs which are not in the European reference panel are coloured grey.

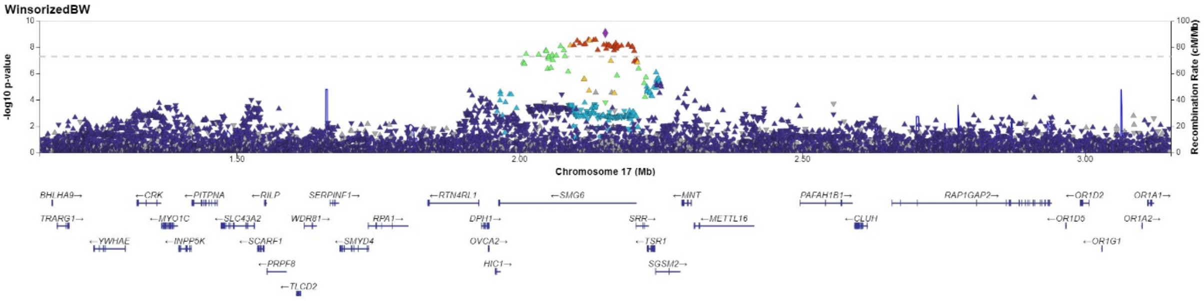

ESM Fig. 35: Locus zoom plot for rs145763145. SNPs which are not in the European reference panel are coloured grey.

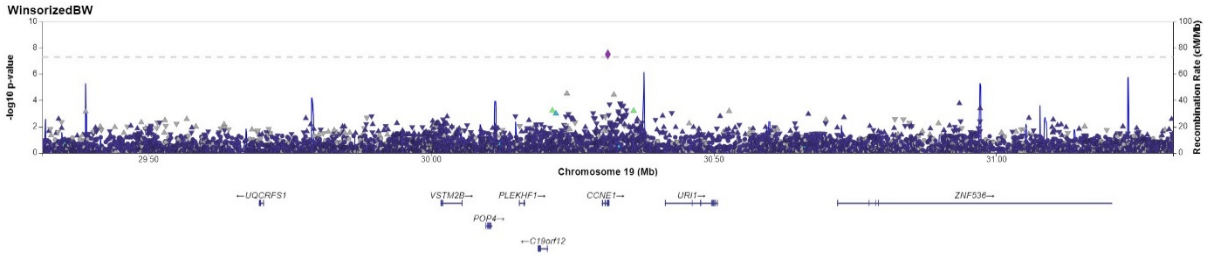

ESM Fig. 36: Locus zoom plot for rs61750863. SNPs which are not in the European reference panel are coloured grey.

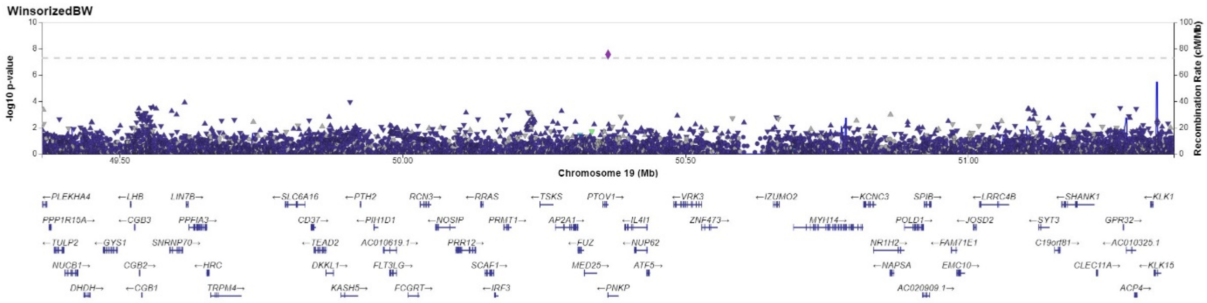

ESM Fig. 37: Locus zoom plot for rs200876443. SNPs which are not in the European reference panel are coloured grey.

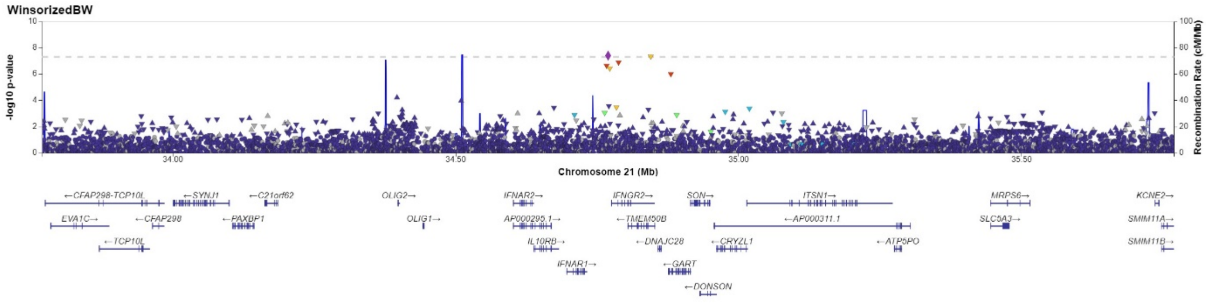

ESM Fig. 38: Locus zoom plot for rs35704817. SNPs which are not in the European reference panel are coloured grey.
